# A Bibliometric Analysis of Research on Ketamine From 2001 to 2020

**DOI:** 10.3389/fnmol.2022.839198

**Published:** 2022-02-24

**Authors:** Huihui Miao, Kang Yu, Danyang Gao, Xiaowan Lin, Ying Cao, Xiao Liu, Hui Qiao, Tianzuo Li

**Affiliations:** Department of Anesthesiology, Beijing Shijitan Hospital, Capital Medical University, Beijing, China

**Keywords:** ketamine, bibliometric analysis, clinical anesthesia, analgesic, depression

## Abstract

**Background:**

Ketamine is an intravenous anesthetic with analgesic effects that has a rapid onset and short duration of action. Many studies have been conducted on the use of ketamine; however, the quantity and quality of such studies have not been reported. Therefore, we aimed to conduct a bibliometric analysis of research on ketamine from 2001 to 2020.

**Methods:**

We used the Web of Science database to get publications on ketamine from January 2001 to December 2020. Various bibliographic information was collected, including the number of publications, year of publication, country of origin, journal name, research hotspots, citation count, and author information.

**Results:**

A total of 5,192 articles were included in the analysis. The United States published the highest number of papers on ketamine and the United States participated in publishing the most papers and disclosure funds. The types of articles in clinical trials were cited more frequently. Most articles on ketamine were published in the journal *Anesthesia and Analgesia*. Furthermore, the antidepressant effect of ketamine has been a research hotspot for the last 20 years.

**Conclusion:**

This study provided a comprehensive analysis of research on ketamine and highlighted the growing interest in ketamine and its antidepressant effects.

## Introduction

Ketamine is a non-competitive ionized N-methyl-D-aspartate (NMDA) receptor antagonist. NMDA receptors are widely present in the central and peripheral nervous systems. Blocking NMDA channels is the main mechanisms of ketamine’ pharmacology effect (Johnson et al., 2015). It is generally believed that ketamine selectively blocks cortical communication system and the thalamo-cortical system, a dissociative anesthesia state in which pain sensation disappears and consciousness may partially exist (Schmid et al., 1999). In addition, ketamine could also promote the endogenous opioid peptides release; affect the metabolism of monoamine neurotransmitters; stimulate μ, δ, and κ opioid receptors; and block Na^+^ and Ca^2+^ plasma channels to exert analgesic effects (Hirota and Lambert, 1996). The effects of ketamine are dose-dependent; in adults, the recovery period after the traditional clinical dose of ketamine for anesthesia is sometimes accompanied by a variety of adverse reactions, such as dreams and hallucinations. Nevertheless, it has become one of the most commonly used basic drugs in pediatric clinical anesthesia because of its convenient route of administration and less respiratory depression; it is often used for pediatric anesthesia and perioperative analgesia. In addition, the intraspinal injection of ketamine as an auxiliary drug has analgesic and preemptive analgesic effects. Besides, the effect and mechanism study of ketamine on antidepressant is increased gradually, therefore, the overview and publication state on ketamine was analyzed in this study.

Bibliometric analyses can evaluate influential papers in a certain field and objectively analyze their study impact. At present, there is no scientific report on the bibliometric analysis of high-quality and highly cited papers on ketamine. The purpose of our research was to investigate the research hotspots and publication trends regarding ketamine, which helps understand its current research status and provides clinicians with accurate medication standards and new ideas for medication. Using bibliometric methods, 5,192 papers on ketamine from 2001 to 2020 were evaluated and their nature, content, and changes over time were analyzed.

## Materials and Methods

### Search Strategy

We used the Web of Science database to investigate publications on ketamine between 2001 and 2020. We used “ketamine” as the search title, limited the article type to “article or review,” and only searched for English publications. We collected the following bibliometric information: year of publication, country, journal, number of citations, authors, funding, disciplines, institutions, and topics. We did not use any exclusion criteria.

### Statistical Analysis

The CiteSpace software was used for bibliometric analysis. Statistical analysis was performed using the SPSS software (version 21.0; IBM Corp., Armonk, NY, United States). The data were expressed as mean (range) or percentage. Categorical and continuous variables were analyzed using the χ^2^-test and independent-sample *t*-test, respectively. Correlation coefficients (r) and *P*-values were calculated using the Spearman’s test. Statistical significance was set at *P* < 0.05.

## Results

### Year and Country of Publication

**FIGURE 1:**
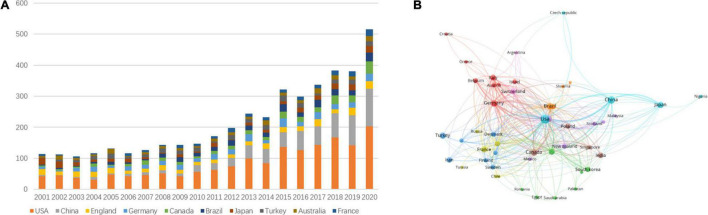
The year and country in which the articles on ketamine were published. **(A)** The number of articles published in different countries each year. **(B)** The country contact map for co-published articles.

In the first 7 years (2001–2007), the number of articles published on ketamine was around 120 per year. From 2008 to 2010, there were no major fluctuations in the number of publications per year. Since 2011, the number of papers published on ketamine has shown an increasing trend (Figure 1A). The year with the largest number of papers published was 2020 (*n* = 515). American authors published the highest number of articles on ketamine (*n* = 1,685), followed by China (*n* = 675) and Germany (*n* = 313). The average number of citations per article published by British authors was 36.55, followed by the United States (34.81) and France (32.54) (Table 1). We also analyzed the cooperation between countries for each published article (Figure 1B) and found that research cooperation was highest with the United States. In addition, there were more papers co-authored by Chinese authors than those of other nationalities.

**TABLE 1:** Number of publications and citations by country.

Countries	Articles	Citations	Average citations per article
United States	1,685	58,655	34.81
China	675	11,101	16.45
Germany	313	8,230	26.29
England	307	11,222	36.55
Canada	262	7,175	27.39
Brazil	256	4,717	18.43
Japan	234	7,013	29.97
Turkey	227	3,413	15.04
France	195	6,345	32.54
Australia	184	4,921	26.74
India	166	2,063	12.43
Italy	144	3,174	22.04
Switzerland	137	3,787	27.64
Iran	117	1,388	11.86
Netherlands	107	4,726	44.17
South Korea	98	1,694	17.29
Poland	85	1,595	18.76
Spain	79	1,253	15.86
Denmark	65	1,774	27.29
New Zealand	64	1,408	22

### Authors and Institutions

**FIGURE 2:**
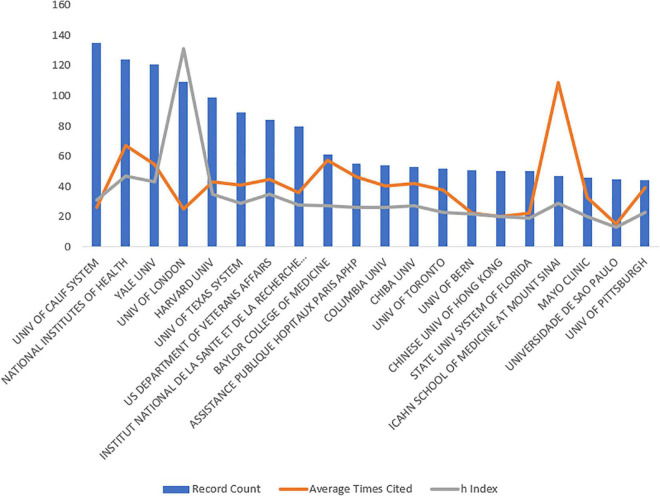
The articles published by different research institutions. The blue bar graph represents the number of articles published by each institution, the red line graph represents the average number of citations per article, and the gray line graph represents the H-index of each institution.

We investigated the top 20 corresponding authors and their institutions according to the number of articles published. The corresponding author with the highest number of publications was Hashimoto Kenji at Chiba University with 46 published papers on ketamine, followed by McIntyre Roger S at the University Health Network in Toronto with 28 publications. We used the H-index to assess the number and level of academic output of the researchers. The highest H-index was that of Hashimoto Kenji (H-index = 24), followed by Zarate Carlos A from the National Institute of Mental Health (H-index = 16). More detailed values are presented in Table 2. Next, we analyzed the top 20 institutions; the institution with the highest number of publications was University of California System (*n* = 135), followed by the National Institutes of Health (*n* = 124) and Yale University (*n* = 121). We also analyzed the H-index for each institution; the University of London had the highest H-index (H-index = 131), followed by the National Institutes of Health (H-index = 47) and Yale University (H-index = 43). More detailed data are presented in Table 3 and Figure 2.

**TABLE 2:** The 20 authors with the highest number of publications.

Author name	Institution	Number of articles	H-index
Hashimoto, Kenji	Chiba University	46	24
McIntyre, Roger S	University Health Network Toronto	28	8
Dahan, Albert	Leiden University Medical Center	22	9
Ning, YuPing	Guangzhou Medical University, Guangzhou Huiai Hospital	22	8
Su, TungPing	National Yang Ming Chiao Tung University	18	9
Yang, JianJun	Nanjing University, Jinling Hospital	18	9
Zarate, Carlos A., Jr.	NIH National Institute of Mental Health	18	16
Thormann, Wolfgang	University of Bern	15	8
Wang, Cheng	US Food & Drug Administration	15	12
Gao, Li	Northeast Agricultural University	14	6
Kanungo, Jyotshna	US Food & Drug Administration	14	9
Morgan, Celia J. A	University of London	14	14
Reus, Gislaine Z	Universidade do Extremo Sul Catarinense	14	9
Zugno, Alexandra I	Universidade do Extremo Sul Catarinense	14	10
Abdallah, Chadi G	Yale University	13	9
Kuo, HannChorng	Buddhist Tzu Chi General Hospital	13	7
Murrough, James W	Icahn School of Medicine at Mount Sinai	13	13
Kabbaj, Mohamed	Florida State University	12	14
Rodrigues, Ana Lucia S	Universidade Federal de Santa Catarina	12	6
Wainer, Irving W	Cooper Medical School of Rowan University	12	10

**TABLE 3:** Top 20 author institutions in terms of number of articles published.

Institution	Articles	Citations	Average citations per article	H-index	Degree centrality
University of California System	135	3,524	26.1	31	234
National Institutes of Health NIH USA	124	8,350	67.34	47	66
Yale University	121	6,592	54.48	43	346
University of London	109	13,1619	25.35	131	90
Harvard University	99	4,279	43.22	35	97
University of Texas System	89	3,633	40.82	29	213
US Department of Veterans Affairs	84	3,781	45.01	35	28
Institut National de la Sante et de la Recherche Medicale Inserm	80	2,896	36.2	28	35
Baylor College of Medicine	61	3,505	57.46	27	139
Assistance Publique Hopitaux Paris Aphp	55	2,553	46.42	26	6
Columbia University	54	2,194	40.63	26	112
Chiba University	53	2,229	42.06	27	62
University of Toronto	52	1,955	37.6	23	258
University of Bern	51	1,146	22.47	22	25
Chinese University of Hong Kong	50	1,003	20.06	20	70
State University System of Florida	50	1,119	22.38	19	31
Icahn School of Medicine At Mount Sinai	47	5,109	108.7	29	173
Mayo Clinic	46	1,494	32.48	20	49
Universidade de São Paulo	45	685	15.22	13	36
University of Pittsburgh	44	1,731	39.34	23	73

### Subjects and Funds

**FIGURE 3:**
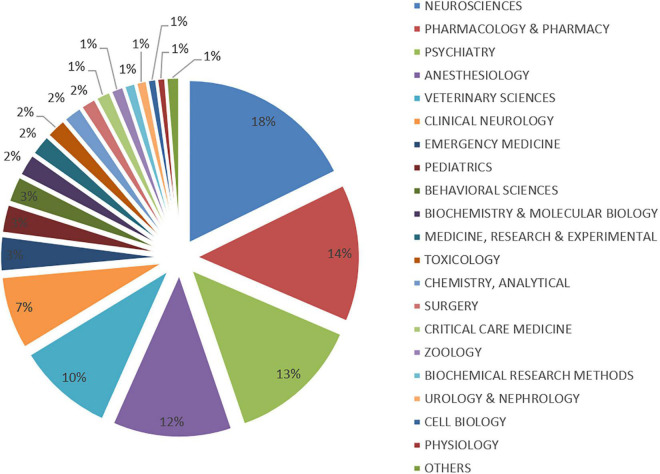
The proportion of articles published in different disciplines.

We analyzed all the journal disciplines that included articles on ketamine in the past 20 years and compiled statistics on these disciplines. We found that most research on ketamine was published in the discipline of neuroscience (*n* = 1,363; 18%) followed by pharmacology and pharmacy (*n* = 1,054; 14%) and psychology (*n* = 1,023; 13%), as shown in Figure 3. We also analyzed the funding agencies mentioned in these articles, and the top 10 funding agencies supporting research on ketamine are shown in Table 4. They included three American institutions, two European, Brazilian, and Japanese institutions, and one Chinese institution. Among them, the United States Department of Health and Human Services (*n* = 637) and National Institute of Health (*n* = 634) funded the maximum number of studies were from the United States, followed by the National Natural Science Foundation of China (*n* = 289).

**TABLE 4:** Top 10 funding agencies with publication volume.

Rank number	Funding agency	Number of publications
1	United States Department of Health Human Services	637
2	National Institutes of Health United States	634
3	National Natural Science Foundation of China	289
4	European Commission	253
5	Conselho Nacional de Desenvolvimento Cientifico e Tecnologico CNPq	103
6	NARSAD	86
7	UK Research Innovation	70
8	Coordenacao de Aperfeicoamento de Pessoal de Nivel Superior CAPES	65
9	Ministry of Education Culture Sports Science and Technology Japan	57
10	Japan Society for the Promotion of Science	45

### Journal Analysis

**FIGURE 4:**
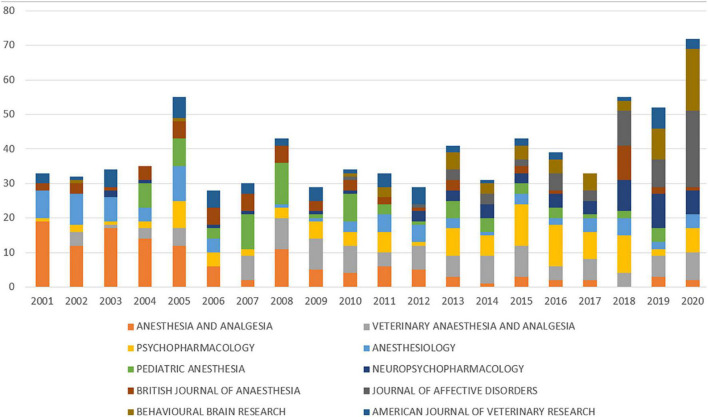
The number of articles published in different journals each year.

Next, we investigated the top 20 journals with articles published on ketamine, as shown in Table 5. The top 20 journals were established by the number of articles on ketamine they published during this period. Among them, the journal with the highest number of articles was *Anesthesia and Analgesia* (*n* = 129; each article was cited 46.39 times on average), followed by the journals *Veterinary Anesthesia and Analgesia* (*n* = 108; each article was cited 13.94 times on average) and *Psychopharmacology* (*n* = 105; each article was cited 4.93 times on average). We quantified the number of publications in various journals per year and found that the number of publications in the journals *Veterinary Anesthesia and Analgesia* and *Behavioral Brain Research* increased every year in the past 10 years. More detailed data are presented in Figure 4.

**TABLE 5:** Ranking of the top 20 journals by citations.

Order	Name	Number of posts	Number of cited	Citations per article	IF	JCR partition
1.	Anesthesia and Analgesia	129	5,984	46.39	5.178	Q1
2.	Veterinary Anesthesia and Analgesia	108	1,506	13.94	1.648	Q2
3.	Psychopharmacology	105	3,673	34.98	4.53	Q2
4.	Anesthesiology	81	4,487	55.4	7.892	Q1
5.	Pediatric anesthesia	75	1,811	24.15	2.556	Q2
6.	Neuropsychopharmacology	68	4,071	59.87	5.251	Q1
7.	British Journal of Anesthesia	58	1,853	31.95	9.166	Q1
8.	Journal of Affective Disorders	58	1,482	25.55	4.839	Q1
9.	Behavioral Brain Research	57	1,279	22.44	3.332	Q2
10.	American Journal of Veterinary Research	56	1,036	18.5	1.156	Q3
11.	Journal of Zoo and Wildlife Medicine	54	425	7.87	0.776	Q3
12.	Neuropharmacology	54	1,664	30.081	5.251	Q1
13.	Pharmacology Biochemistry and Behavior	54	1,197	22.17	3.533	Q2
14.	PLoS ONE	54	1,242	23	3.24	Q2
15.	Biological Psychiatry	49	5,991	122.27	13.382	Q1
16.	Acta Anaesthesiologica Scandinavica	48	1,438	29.96	2.105	Q4
17.	American Journal of Emergency Medicine	47	999	21.26	2.469	Q2
18.	Journal of Psychopharmacology	47	1,502	31.96	4.153	Q2
19.	Neuroscience Letters	47	692	14.72	3.046	Q3
20.	International Journal of Neuropsychopharmacology	43	1,942	45.16	5.176	Q1

### Citations

**FIGURE 5:**
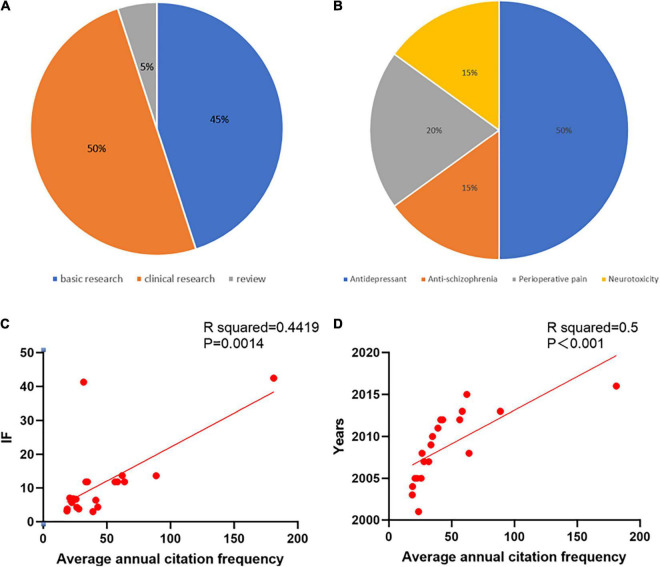
The classification and correlation analysis of the top 20 cited articles on ketamine. **(A)** The proportions of the top 20 ketamine research article classifications. **(B)** The proportions of the research content categories of the top 20 cited articles on ketamine. **(C)** There was a significant correlation between the average number of citations per year and the impact factor based on the correlation analysis (R squared = 0.4419; *P* = 0.0014). **(D)** There was a significant correlation between the average number of citations per year and the year of the analysis (R squared = 0.5; *P* < 0.001).

In general, the number of citations varied, and we identified the 20 most cited articles. These 20 articles included 9 basic research, 1 review, and 10 clinical research articles. Based on the effects of ketamine, we classified the research content of these articles as follows: 10 articles, antidepressant effects; three articles, antischizophrenic effects; four articles, effects on perioperative pain; and three articles, effects on neurotoxicity (Figures 5A,B). The top three cited articles were “Cellular mechanisms underlying the antidepressant effects of ketamine: Role of alpha-amino-3-hydroxy-5-methylisoxazole-4-propionic acid receptors,” (766 citations) (Maeng et al., 2008); “NMDAR inhibition-independent antidepressant actions of ketamine metabolites,” (724 citations) (Zanos et al., 2016); and “Antidepressant efficacy of ketamine in treatment-resistant major depression: A two-site randomized controlled trial,” (621 citations) (Murrough et al., 2013). We found that the top four cited papers were all related to the antidepressant effects of ketamine, and ten out of these twenty articles were on the antidepressant mechanism and clinical applications of ketamine (Table 6).

**TABLE 6:** Top 20 cited articles.

Rank number	Topic	Corresponding author	Institution	Journal	Year	Cited frequency
1	Cellular mechanisms underlying the antidepressant effects of ketamine: Role of alpha-amino-3-hydroxy-5-methylisoxazole-4-propionic acid receptors	Manji, HK	NIH, Lab Mol Pathophysiol and Expt Therapeut	Biological Psychiatry	2008	766
2	NMDAR inhibition-independent antidepressant actions of ketamine metabolites	Gould, TD	University of Maryland School of Medicine Department of Psychiatry	Nature	2016	724
3	Antidepressant Efficacy of Ketamine in Treatment-Resistant Major Depression: A Two-Site Randomized Controlled Trial	Mathew, SJ	Icahn Sch Med Mt Sinai	American Journal of Psychiatry	2013	621
4	Replication of Ketamine’s Antidepressant Efficacy in Bipolar Depression: A Randomized Controlled Add-On Trial	Zarate, CA	NIH, Department of Health and Human Services	Biological Psychiatry	2012	451
5	Effects of ketamine in normal and schizophrenic volunteers	Lahti, AC	University of Maryland School of Medicine Department of Psychiatry Research Center	Neuropsychopharmacology	2001	448
6	Ketamine-induced loss of phenotype of fast-spiking interneurons is mediated by NADPH-oxidase	Behrens, MM	University of California San Diego, Department of Medicine	Science	2007	413
7	Rapid and Longer-Term Antidepressant Effects of Repeated Ketamine Infusions in Treatment-Resistant Major Depression	Murrough, JW	Mount Sinai School of Medicine	Biological Psychiatry	2013	409
8	Remifentanil-induced postoperative hyperalgesia and its prevention with small-dose ketamine	Chauvin, M	Assistance Publique Hôpitaux de Paris	Anesthesiology	2005	386
9	Effects of Intravenous Ketamine on Explicit and Implicit Measures of Suicidality in Treatment-Resistant Depression	Price, RB	Rutgers, The State University	Biological Psychiatry	2009	369
10	Ketamine-induced neuronal cell death in the perinatal rhesus monkey	Slikker, W	U.S. Food and Drug Administration’s National Center for Toxicological Research	Toxicological Sciences	2007	363
11	Ketamine anesthesia during the first week of life can cause long-lasting cognitive deficits in rhesus monkeys	Paule, MG	U.S. Food and Drug Administration’s National Center for Toxicological Research	Neurotoxicology and Teratology	2011	351
12	Safety and Efficacy of Repeated-Dose Intravenous Ketamine for Treatment-Resistant Depression	aan het Rot, M	University of Groningen	Biological Psychiatry	2010	347
13	Signaling pathways underlying the rapid antidepressant actions of ketamine	Duman, RS	Yale University	Neuropharmacology	2012	343
14	Ketamine and postoperative pain—a quantitative systematic review of randomized trials	Elia, N	University Hospitals Geneva	Pain	2005	337
15	Ketamine use: a review	Curran, HV	UCL, Clinical Psychopharmacology Unit	Addiction	2012	330
16	Acute administration of ketamine induces antidepressant-like effects in the forced swimming test and increases BDNF levels in the rat hippocampus	Quevedo, J	Universidade do Extremo Sul Catarinense	Progress in Neuro-Psychopharmacology and Biological Psychiatry	2008	317
17	Effects of ketamine and N-methyl-D-aspartate on glutamate and dopamine release in the rat prefrontal cortex: Modulation by a group II selective metabotropic glutamate receptor agonist LY379268	Lorrain, DS	Merck Research Laboratories	Neuroscience	2003	317
18	Potential of ketamine and midazolam, individually or in combination, to induce apoptotic neurodegeneration in the infant mouse brain	Olney, JW	Washington University	British Journal of Pharmacology	2005	312
19	Ketamine and Other NMDA Antagonists: Early Clinical Trials and Possible Mechanisms in Depression	Nemeroff, CB	University of Miami	American Journal of Psychiatry	2015	310
20	Ketamine as adjuvant analgesic to opioids: A quantitative and qualitative systematic review	Subramaniam, K	Harvard University	Anesthesia and Analgesia	2004	301

In addition, we analyzed the correlation between the average number of citations, year of publication, and impact factors of the 20 most cited journals; the average number of citations, year of publication (*R* = 0.5, *P* < 0.001, and impact factor (*R* = 0.4419, *P* = 0.0014) were all significantly correlated with one another (Figures 5C,D).

### Research Hotspots and Publication Trends

**FIGURE 6:**
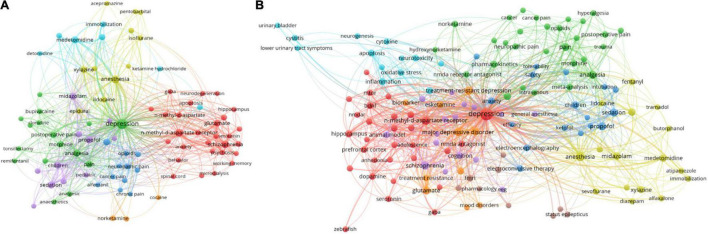
A summary of the hotspot trends of articles on ketamine from 2001 to 2020. The size of the circles and the thickness of the line represent the frequency of co-occurring keywords. **(A)** Hotspots of articles published on ketamine from 2001 to 2010. **(B)** Hotspots of articles published on ketamine from 2011 to 2020.

Research hotspots were identified by the frequency of two keywords that appeared together in the same publication. Additionally, the size of the circles and the thickness of the line represented the frequency of co-occurrence of the keywords. We hypothesized that the hotspots in research on ketamine changed with time; therefore, we classified and summarized all the literature research hotspots every 10 years. From 2001 to 2010, the research interest on ketamine was more about the mechanism of action in neuropathy or brain function, as shown in the red cluster. “Depression” was associated with anesthesia or analgesia, as shown in the green cluster (Figure 6A). We found that the research hotspots of articles on ketamine was higher from 2010 to 2020 than in the previous 10 years. “Depression” was the most frequently encountered keyword that appeared with the mechanism cluster (in red), indicating a greater focus on identifying the molecular targets. Our search statistics on the topic of articles in the past 10 years also confirmed that the antidepressant effect of ketamine was the focus of these articles (Figure 6B).

## Discussion

In this study, we searched for articles on ketamine published in the Web of Science database from 2001 to 2020 and analyzed their basic information. We also conducted a correlation analysis of the articles’ citation frequencies. To avoid the differences caused by the year of publication of the article, we chose the average annual citation frequency as a reference indicator and performed a correlation analysis between the year of publication and the impact factor of the articles. The correlation analysis revealed that the articles published later had a higher citation frequency and impact factor. This indicates that ketamine has received increasing attention in recent years with higher numbers of open-access publications on it. Finally, the hotspot trend analysis indicated that an increasing amount of research on ketamine in the past 10 years has focused on its mechanism as an antidepressant.

The journals wherein articles on ketamine were published gradually changed from *Anesthesia and Analgesia* and *Anesthesiology* to *Behavioral Brain Research* and *Veterinary Anesthesia and Analgesia*. This could be attributed to the fact that ketamine has become a hot topic of research in recent years owing to its antidepressant effects and multiple animal experiments involving it. In terms of co-authorship (Supplementary Figure 1), we found that Rosenblat, Joshua D, Nasri, Flora, and Iee, Yena et al. co-authored more articles on ketamine, which may be since they are from the same institution or from the same country. Among the cooperative institutional relationships (Supplementary Figure 2), University of California System and university of texas system take great part and co-operative with other institutions frequently. Some other institutions include Guangzhou Medical University, Chiba University, Chinese University of Hong Kong, and Nanjing University were also co-operative closely. The amount of cooperation between these institutions is much higher than that of other institutions, and the number of publications of these institutions is also higher than that of other institutions, which is consistent with our analysis results. By analyzing the correspondence between authors and institutions, we found that Hashimoto, the author with the largest number of papers, and Chiba University, the institution where Kenji works, also published more papers (*n* = 53), ranking 12th, and the author Morgan, who was the 12th author with the same total number of papers. The University of London, where Celia J. A belongs, ranks fourth and Yale University, where the authors of the 15th publishing volume, Abdallah and Chadi G, ranks third in terms of total publication volume. This shows that the institutions of authors with high publication volumes tend to have higher publication volumes, and these authors and institutions are more willing to collaborate with other institutions on co-authoring articles.

In recent years, ketamine has received considerable attention in the treatment of clinical depression, which has aroused a conventional drug in new use; However, this renewed attention might be partly due to an increase in the number of patients with depression in recent years. In addition, more financial disclosures have reflected state and government support for this research. With an aging population, the application and side effects of clinical anesthetics will continue to attract attention. The continuous progress of ketamine research and the strong support of the government has facilitated the developments in the field of anesthesia to a certain extent.

Depression is a common mental illness. Existing antidepressants have a slow onset of action, usually 3–4 weeks, and the failure rate is high (up to 40%) (Thase et al., 2005; Krishnan and Nestler, 2008). Therefore, rapid-acting and effective antidepressants need to be developed; this is a medical problem that needs to be solved urgently. Professor Krystal from the Department of Psychiatry at Yale University School of Medicine and others reported for the first time in 2000 and found that ketamine had a rapid antidepressant effect (Rmbab et al., 2000). A single intravenous infusion of ketamine (0.5 mg/kg) produced an effective antidepressant effect in 4 h and lasted for at least 72 h. Zarate et al. (2006) conducted another randomized double-blind controlled study on patients with refractory depression using the same method of administration and dose, and the results showed that intravenous infusion with a sub-anesthetic dose (0.5 mg/kg) of ketamine improved the symptoms of depression significantly in 110 min after administration. About 71% of the patients showed a significant improvement in their depression symptoms, and 29% of the patients felt relieved 1 day after the administration (Zarate et al., 2006). Price et al. (2009) once again confirmed the rapid and effective antidepressant effect of ketamine and found that it can effectively alleviate or eliminate suicidal ideation in depression patients within 24 h after administration. From 2010 to 2015, Professor Zarate’s research team reported a series of research results on the clinical efficacy of ketamine as an antidepressant. These results showed that ketamine could produce rapid, effective, and long-lasting antidepressant effects (Zarate et al., 2012; Ionescu et al., 2015; Moaddel et al., 2015); ketamine not only quickly alleviated the patients’ depression symptoms but also attenuated their suicidal tendencies (Ballard et al., 2014). The antidepressant effect of ketamine was first discovered in the clinic, followed by many animal experiments to explore the molecular targets of ketamine and the related mechanism of action. Ketamine has been demonstrated to have significant antidepressant effects in a variety of classic depression models (Maeng et al., 2008; Li et al., 2010; Autry et al., 2011; Beurel et al., 2011; Xu et al., 2013; Zhou et al., 2015).

Several highly cited studies discussed the mechanism and duration of ketamine’ antidepressants effect. The study by Maeng et al. (2008) hypothesized that a-amino-3-hydroxy-5-methylisoxazole-4-propionic acid (AMPA) receptor throughput facilitated ketamine’ antidepressant effects. Ketamine was administered at doses of 0.5, 2.5, and 10 mg/kg, and single injections of ketamine can produce rapid antidepressant effects (Maeng et al., 2008). For the persistence of the antidepressant effect of ketamine, Maeng et al. (2008) treated mice with saline, ketamine (2.5 mg/kg) and imipramine (20 mg/kg) and found that only mice in the ketamine group had lower immobility after 2 weeks, suggesting that the ketamine’ antidepressant effect lasted for a fortnight. The mice were then fear-trained and treated with saline and ketamine and it was found that ketamine did not result in memory impairment. The duration of ketamine induced immobility was shortened after the use of AMPA receptor antagonists. The use of AMPA antagonists significantly blocked the antidepressant effects of MK-801 (a non-selective NMDA antagonist) and Ro25-6981 (a selective NR2B antagonist), and interestingly, neither of them had as long-lasting antidepressant effects as ketamine. Zanos et al. (2016) elaborated that the antidepressant actions of ketamine was NMDA receptors independently, but with AMPA receptor activated. Zanos et al. (2016) also demonstrated the importance of the ketamine metabolite (2S,6S;2R,6R)-hydroxynorketamine (HNK) in the antidepressant effect at the molecular level. Compared with (2S,6S)-HNK from (S)-ketamine, (2R,6R)-HNK derived from (R)-ketamine established more potent antidepressant effect and showed a more pronounced dose dependence than S-ketamine, and that (2R,6R)-HNK showed no significant toxic effects compared to direct ketamine administration, suggesting R-ketamine will be more benefit as a new type of antidepressant drug. In the clinical randomized, double-blind add-on trial, Carlos reported an improvement in depressive symptoms within 3 days after the administration of 0.5 mg/kg ketamine injection for bipolar depression patients, with the most significant side effect being dissociative symptoms, which occurred 40 min after injection (Zarate et al., 2012). In another high-impact clinical research by Murrough et al. (2013) who used 0.5 mg/kg ketamine or 0.045 mg/kg midazolam single infusion in treatment-resistant major depression patients and showed that ketamine had a faster onset of action (dominance ratio 2.18) and higher efficacy (64% for ketamine; 28% for imipramine). Therefore, the difference in study design, dose or disease states did not affect ketamine’s antidepressant effect.

Through statistical analysis, we found that more ketamine articles are published in neuroscience. The possible reason is that many basic researches on ketamine have been published in large quantities, to figure out the mechanism of ketamine. The following disciplines are pharmacology and psychology list second and psychiatry list third, due to the large number of published studies on the antidepressant effects of ketamine. The focus of different disciplines may also different. For example, the focus of anesthesia may be the application of anesthesia and the use of anesthesia techniques in clinical practice, while the focus of pharmacology maybe the pharmacological effects, toxicological effects, and half-life of drugs themselves. However, some of these disciplines like anesthesiology or neurosciences also intersect with each other, which can participate in both intraoperative anesthesia induction and antidepressant study of ketamine. The differences of topic hotspots or focus keywords among different disciplines would be analyzed in the further study.

Our bibliometric analysis had some inherent limitations. First, some recently published high-impact articles were not included in the “20 most cited articles” because they were not cited sufficiently. However, this does not mean that these articles were not important. Second, we found that articles published in journals with high impact factors tended to receive more attention. In the correlation analysis, we found that the impact factor and the average number of citations of the journal were positively correlated, which showed that high impact factors can inherently lead to bias.

## Conclusion

We searched and analyzed 5192 English articles published on ketamine from 2001 to 2020. Despite some limitations, our study has found that research interest in ketamine has gradually increased. The research was mostly focused on the clinical application and mechanism of ketamine as an antidepressant, which has also led to more publications on ketamine in mental illness and veterinary journals. Moreover, basic scientific research on the antidepressant mechanism of ketamine has evidently in the past 10 years.

## Data Availability Statement

The original contributions presented in the study are included in the article/Supplementary Material, further inquiries can be directed to the corresponding author/s.

## Author Contributions

TZL and HQ conceived, designed the structure of this manuscript, and revised the manuscript. HHM, KY, DYG, XWL, YC, and XL analyzed and wrote the manuscript. All authors contributed to the article and approved the submitted version.

## Conflict of Interest

The authors declare that the research was conducted in the absence of any commercial or financial relationships that could be construed as a potential conflict of interest.

## Publisher’s Note

All claims expressed in this article are solely those of the authors and do not necessarily represent those of their affiliated organizations, or those of the publisher, the editors and the reviewers. Any product that may be evaluated in this article, or claim that may be made by its manufacturer, is not guaranteed or endorsed by the publisher.
