# Supplementary material for: A Bibliometric Analysis of Research on Ketamine From 2001 to 2020
Source: Front Mol Neurosci. 2022 Feb 24;15:839198. doi: 10.3389/fnmol.2022.839198 (PMC8908203; doi:10.3389/fnmol.2022.839198)
Supplement: Supplementary file 1 [file Image_1.PDF]

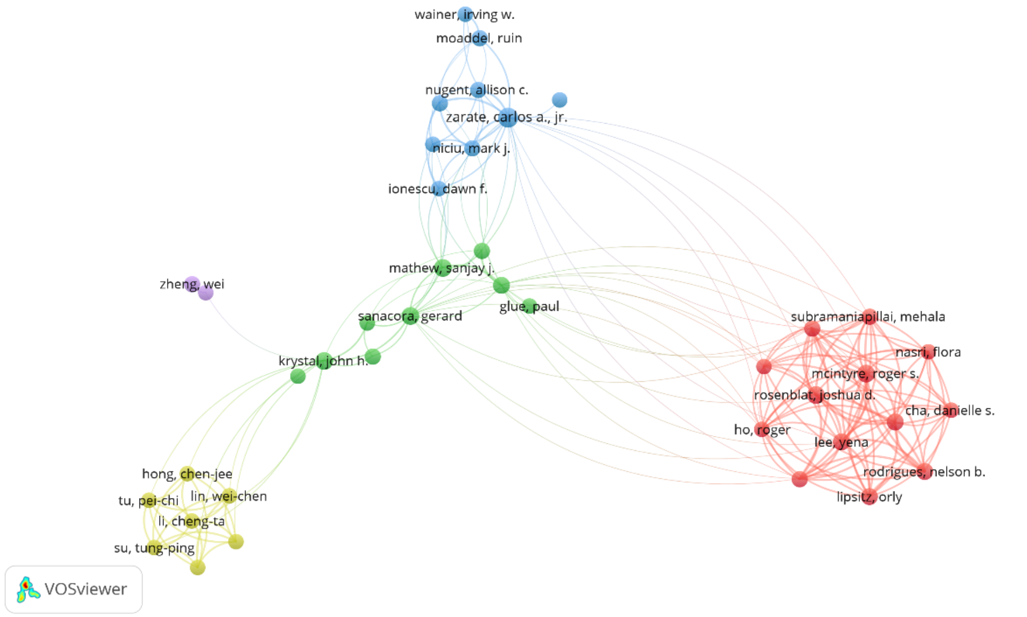

Supplementary figure 1. The co-operation of the authors on ketamine

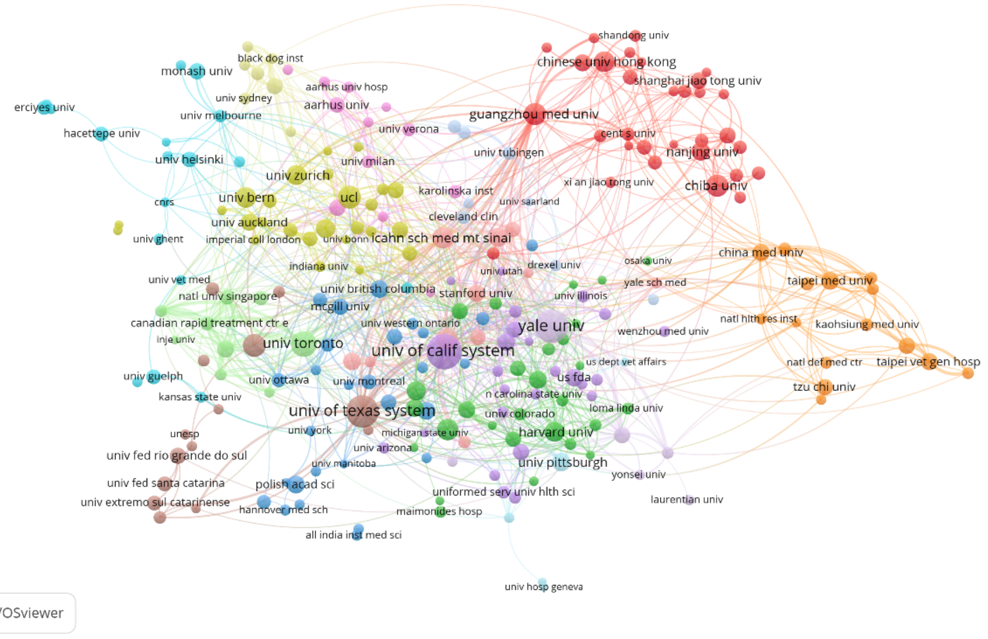

Supplementary figure 2. The co-operation of the intuitions on ketamine
